# Socioeconomic status and substance use among Swiss young men: a population-based cross-sectional study

**DOI:** 10.1186/s12889-016-2949-5

**Published:** 2016-04-14

**Authors:** Eleni Charitonidi, Joseph Studer, Jacques Gaume, Gerhard Gmel, Jean-Bernard Daeppen, Nicolas Bertholet

**Affiliations:** Doctoral School, Faculty of Biology and Medicine, University of Lausanne, Lausanne, Switzerland; Alcohol Treatment Center, Department of Community Medicine and Health, Lausanne University Hospital, Beaumont 21b, P2, 02, 1011 Lausanne, Switzerland

**Keywords:** Socio-economic status, Substance use, Alcohol, Tobacco, Illicit drugs

## Abstract

**Background:**

Socioeconomic status (SES) is often inversely related to health outcomes and is likely to play a role in the use of psychoactive substances among young individuals, although little consensus exists on the association between SES and substance use.

The purpose of the study was to determine the association of three SES indicators (perceived family income, education level of participants, and parental education level) with past year use of alcohol, tobacco, cannabis, other illicit drugs and non-medical use of prescription drugs (NMPD) among Swiss young men.

**Methods:**

Population-based cross-sectional study of 5,702 men at mean age twenty. Associations between SES indicators and substance use were assessed with regression models adjusted for age and linguistic region.

**Results:**

Participants with average or below average perceived family income were less likely to report any use of alcohol (OR = O.75) but more likely to use tobacco daily (OR = 1.31) and cannabis weekly (OR = 1.27) compared to those with perceived above average family income.

Participants whose parents had only achieved obligatory education were less likely to engage in any use of alcohol (OR = 0.30), monthly risky single occasion drinking (RSOD, defined as 6 or more drinks per occasion) (OR = 0.48), any use of cannabis (OR = 0.53) and other illicit drugs (OR = 0.58), whereas those whose parents had only achieved secondary education were less at risk of engaging in cannabis (OR = 0.66 for any use and OR = 0.77 for more than once a week use) and other illicit drugs (OR = 0.74) use, compared to those whose parents had achieved tertiary education. Compared to participants who completed secondary or tertiary education, those who completed only obligatory education reported a higher risk of tobacco (OR = 1.18 for any use, OR = 1.31 for daily use), cannabis (OR = 1.23 for any use, OR = 1.37 for more than once a week use), and other illicit drugs (OR = 1.48) use. No association was found between NMPD and the studied SES variables.

**Conclusion:**

The relationship between SES and substance use was complex in this sample. Higher socioeconomic status was associated with more alcohol and other illicit drugs use, while lower socioeconomic status was related to more tobacco use. Education level and perceived family income may have different impacts on substance use and may vary by substance.

## Background

The impact of socioeconomic status (SES) on health behaviors and other outcomes has been the centerpiece of several studies [1–9]. It has been shown that death rates are higher in lower SES groups in all European countries [10–12]. Furthermore, there is growing interest in the association of SES with health-related behaviors in early adulthood, but there is conflicting evidence regarding the association of SES with substance use in this age group. The social gradient appears to differ when different indicators of SES are examined. For example, some studies have shown that some indicators of higher SES, such as parental education or family income, are associated with more use of substances [13–15], while own education level is associated with less use of substances [14]. The social gradient for a particular SES indicator may also differ across substances, since some studies have found that high SES indicators are associated with more alcohol consumption, but with less use of other substances such as tobacco [15]. Alternatively, other researchers report no significant association of different SES indicators with substance use [16]. To our best knowledge, there is no available data relating SES indicators to the non-medical use of prescription drugs.

Thus, current evidence linking SES to substance use has been mixed. Associations can differ according to the various SES indicators, such as own or parental education level, family income or perceived social position, as well as by substance used. Studying substance use across socio-economic strata will assist policy makers and public health agencies in refining preventive interventions that target substance use and abuse. The identification of inequalities in the distribution of substance use across socio-economic strata will add to the body of evidence suggesting a social gradient in health. It will inform on which population subgroups are more likely to use which substance, and indicate who should be targeted with preventive interventions. It is important to assess whether the same associations between substance use and SES indicators can be found for all substances. If one substance is not following a social gradient, this will call for preventive strategies targeting socio-economic subgroups differently for each substance. The research herein presents in detail the associations of three SES indicators (perceived family income, own education and parental education) with substance use in a sample of young men from the Swiss general population.

The first aim was to determine whether SES indicators among young adults are associated with past year prevalence of alcohol, tobacco, cannabis, and other illicit drug use, as well as non-medical use of prescription drugs (NMPD). The second aim was to examine the associations of SES indicators and intensity of substance consumption, i.e., monthly risky single occasion drinking (monthly RSOD, defined as drinking 6 or more drinks per occasion at least once a month), daily use of tobacco and more than weekly use of cannabis.

## Methods

### Study population and setting

The current cross-sectional study was conducted in Switzerland as part of the Cohort Study on Substance Use Risk Factors (C-SURF) [17, 18]. The participants in this large study are young Swiss men who were approached and recruited during the mandatory two-day assessment procedure to determine eligibility for military service. Because it is mandatory, virtually all males at age 19 must attend one of the recruitment centers, thus providing a unique opportunity to access a large representative sample of the general population of young men in Switzerland. Recruitment for C-SURF took place at three of the six army centers, one in the French-speaking sector (Lausanne) and two in the German-speaking sectors (Windisch and Mels). These three centers are in charge of assessing the eligibility for Swiss military service in 21 of the 26 cantons of Switzerland.

Although women are allowed to voluntarily join the military service, most do not, thus they would not represent the general population and were excluded from C-SURF. Because substance use may influence eligibility in the army, there is some risk of under- or over-reporting substance abuse among attendees of the recruitment centers. Therefore all potential research participants were assured that any and all information gathered as part of the study was to be kept confidential from the army, and could not affect conscription procedures. Strong efforts were made to ensure that participants understood that the research was entirely independent of the army, which in no way could gain access to data connected to any individual.

All conscripts attending the centers were eligible for participation in C-SURF, provided they furnished written informed consent. Research staff informed potential participants (conscripts) that the study was a longitudinal survey. The goal of the study, studying substance use risk factors, was made clear to potential participants. Potential participants were also informed on the study procedures and the right to withdraw from the study at any time without penalty. Participants completed informed consent at the study recruitment site (army recruitment centers). The study questionnaires were completed by the participants after they left the army recruitment centers. The study was approved by the Ethics Committee for Clinical Research at the Lausanne University Medical School (protocol number 15/07).

Recruitment for this study took place between August, 2010 and November, 2011, during which time 13,245 center attendees were offered participation. Conscripts have the option of attending the recruitment centers while younger than 19, explaining some variation in age ranges observed in other studies conducted by our group at the army recruitment centers [19–21]. The 7,563 conscripts who were willing to participate received research questionnaires after leaving the recruitment centers, either in paper-pencil or online form, according to their preference.

### Dependent variables

#### Alcohol use

Alcohol use in the past 12 months was assessed with drinking frequency and quantity questions: *”How often do you usually drink alcohol?”* (participants were offered a range of response options: 7, 6, 5, 4, 3, 2, 1 day(s) a week; 2–3 times a month; once a month or less; never) and, *“How many standard drinks do you drink on average on days when you drink alcohol?”*(open question) [22]. A standard drink was defined as 100 ml of wine, 250 ml of beer, 275 ml of a premixed drink containing spirits or 20 ml of spirits (each ∼ 10 g of ethanol), which is the commonly used definition of a standard drink in Switzerland [23]. The questionnaire included visual aids with pictures of standard drinks and labels so as to identify various container sizes.

The frequency of risky single occasion drinking (RSOD), defined as 6 or more drinks per occasion, was assessed with the following question: *“How many times have you had 6 or more standard drinks with alcohol, during the same occasion”* (Participants were offered a range of response options: every day or nearly every day; every week; every month; less than once a month, never)*.* The time reference was the past 12 months [24]. Participants were later grouped into two categories: presence of monthly RSOD (every day or nearly every day, every week, every month) or absence of monthly RSOD (less than once a month, never). In Switzerland, six drinks contain ∼ 60 g of pure alcohol and is equivalent to the commonly used US measure of 5+ drinks of 12 g per drink [25]. Our definition of RSOD is equivalent to the definition of NIAAA as well as to European standards.

Four dependent variables reflecting alcohol use over the past 12 months were used: 1) Any alcohol use over the past 12 months; 2) Presence of monthly RSOD; 3) Presence of weekly risky drinking (defined as 21 standard drinks per week or more); and 4) Weekly average consumption (mean number of standard drinks per week).

The weekly risky drinking definition used in the present study approximates the World Health Organization and other European clinical guidelines of risky drinking and the US National Institute on Alcohol Abuse and Alcoholism definition [18, 21, 23].

#### Tobacco use

Tobacco use was measured as any cigarette use during the past 12 months through a yes or no question (*“Have you smoked cigarettes in the past 12 months?”),* as well as the frequency of smoking, through the question “*How often have you generally smoked cigarettes in the past 12 months?”*(Participants were offered a range of 6 response options, from “every day” to “once in a month or less”). Participants were later grouped into two categories: daily use, or less*.* Thus, the two tobacco measures used as dependent variables were any use over the past 12 months and daily tobacco use.

#### Cannabis use

Cannabis use was assessed with questions included in the Cannabis Use Disorders Identification Test [26]. We identified participants reporting any use over the past 12 months and differentiated those who consumed once a week or less from those who consumed more than once a week using the following questions: *“Have you used any cannabis over the past 12 months?”*(yes or no question) and*, “How often have you used cannabis over the past 12 months?”*(with 5 response options from “monthly or less” to “every day or almost every day*”*).

#### Other illicit drug use

Participants were asked about their use over the past 12 months use of the following substances: ecstasy; cocaine; heroin; and magic mushrooms through the question *“Have you ever taken any of these drugs in the past 12 months? If yes, how often?”*(with 3 response options “never”, “1 to 3 times” and “4 times or more”).

### Non-medical use of prescription drugs (NMPD)

Participants were asked about their use on any day over the last 12 months of sleeping pills, tranquilizers, painkillers, stimulants, antidepressants, and beta blockers *(“Now we would like to ask you about your experience with prescribed medicine in the last 12 months that you may have decided to use of your own will-that is, either without a doctor’s prescription or without a doctor telling you to use them. People use the following medicine or drugs of their own will to feel more alert, to relax or calm down, to feel better, to enjoy themselves, or to get high or just to see how they would work. Have you taken such medicine of your own will?”).* Participants were offered 8 response options, varying from “never” to “4 times a week or more”.

### Independent variables

#### Socioeconomic status (SES) indicators

We used three indicators of participant SES: perceived family income, own education level, and parental education level. Perceived family income was assessed because there is growing evidence that subjective economic power is a better predictor of health outcomes than objective economic power [27–29]. Hence, participants were asked to report their perception of their family income *(“How well off is your family compared to other families in your country?”*). Participants were grouped in two categories: average or below average, and above average. Participant education level was determined as the highest education level achieved by the participants at the time of assessment, characterized as having received obligatory or elementary vocational education only or having received more than that. Parental education level was assessed by asking participants to report the highest education level achieved by their parents (either father or mother) *(“What is the highest level of education your parents achieved?”*). Parental education level was categorized into obligatory, secondary (vocational training or school) or tertiary (university or university of applied sciences).

### Analyses

Relationships between SES indicators and substance use were first assessed using chi-square tests except for number of drinks/week for which analysis of variance testing equality of means was used. Subsequently, linear and logistic regression models adjusted for age, living environment (rural, i.e. < 10000 inhabitants vs. urban, i.e. ≥ 10000 inhabitants) and linguistic region (German—vs. French-speaking) were fit. In our sample, the age range was 17.9–27.8. In Switzerland, the legal age for purchase of beer and wine is 16, and 18 for hard liquors. For tobacco, the legal age for purchase varies by Canton (from no restriction to 18 years old, with most Cantons having a 16 or 18 year old legal age). Cannabis is an illegal drug in Switzerland (as the other drugs studied). Substance use is known to be associated with age. In addition, age can be associated with SES, notably the highest achieved education level. Analyses were therefore adjusted for age. Analyses were also adjusted for linguistic region and living environment since these can be associated with both SES indicators and substance use patterns. Substance use outcomes were first regressed on each SES indicators separately. Then, all SES indicators were tested simultaneously in a fully adjusted model. Finally, in order to test whether the associations of each SES indicators was moderated by other SES indicators, all two-way and three-way interactions between SES indicators were entered in the fully adjusted model. Since none of the interactions reached significance, results are not reported. In the regression models, SES indicators were contrasted as follows: average or below average vs. above average for perceived family income, obligatory or elementary vocational education only vs. more than obligatory or elementary vocational education for own education level, and tertiary vs. obligatory and secondary education for parental education level.

The dependent variables were any use of alcohol (past 12 months), prevalence of monthly risky single occasion drinking (RSOD), prevalence of weekly risky drinking, number of drinks per week, prevalence of tobacco use, cannabis use, other illicit drugs use (ecstasy, cocaine, heroin and mushrooms), as well as NMPD use (sleeping pills, tranquilizers, painkillers, stimulants, antidepressants, beta blockers).

## Results

Data were collected between September 2010 and March 2012. A total of 5990 participants filled in the baseline questionnaire, of which 5,702 completed the questions on SES and were included in the present study. Details concerning the differences between consenters vs. non-consenters, and responders vs. non-responders have been reported elsewhere [17, 18]. Table 1 presents demographic characteristics of the sample and socioeconomic status, perceived family income, own highest achieved education level and highest achieved education level of parents. A little less than half (44.5 %) of the participants reported above average perceived family income, 50.1 % had completed obligatory or elementary vocational education and 49.9 % had higher than obligatory education. Highest achieved parental education level was mostly secondary (50.8 %) and tertiary (42.9 %). A vast majority (92.3 %) of conscripts reported having used alcohol over the past 12 months, and nearly half of these (46.2 %) reported monthly RSOD, but only a small number of participants (6.2 %) engaged in weekly risky drinking (21 or more drinks per week). Tobacco was used by 47.2 % of the sample, including 21.0 % who reported daily use; 30.6 % of the participants reported any use of cannabis during the past year, and 9.5 % of these used it more than once a week. During the past year, only 6.3 % had consumed at least one other illicit drug and 10.6 % had taken at least one NMPD. Other than cannabis, the most frequently used illicit drug was ecstasy (3.7 %), while painkillers were the most frequently used NMPD (6.8 %).

**Table 1:** Descriptive characteristics of the sample (*N =* 5702)

	N/Mean	%/SD
Age	19.99	1.23
Linguistic region		
German-speaking	2570	45.1
French-speaking	3132	54.9
Living environment		
Rural (<10000 inhabitants)	3440	60.3
Urban (≥ 10000 inhabitants)	2262	39.7
Perceived family income		
Average or below average	3164	55.5
Above average	2538	44.5
Highest achieved own education level		
Obligatory or elementary vocational education	2858	50.1
Higher than obligatory or elementary vocational education	2844	49.9
Parents’ highest achieved education level		
Obligatory	362	6.3
Secondary	2894	50.8
Tertiary	2446	42.9
Alcohol use		
Any use (past 12 months)	5265	92.3
RSOD monthly or more^a^	2635	46.2
Weekly risky drinking^b^	355	6.2
Number of drinks/week (mean SD)	7.37	13.14
Tobacco use		
Any use (past 12 months)	2693	47.2
Daily use	1196	21.0
Cannabis use		
Any use (past 12 months)	1744	30.6
More than once a week	543	9.5
Other illicit drugs use^c^	358	6.3
Any use (past 12 months)		
NMPD use^d^	607	10.6
Any use (12-month use)		

*Note:* ^a^Risky single occasion drinking (RSOD) defined as drinking 6 or more drinks on one occasion. ^b^Weekly risky drinking, defined as drinking 21 drinks per week or more. ^c^Ecstasy or cocaine or heroin or magic mushroom. ^d^Sleeping pills or tranquilizer or painkiller or stimulant or antidepressant or beta-blocker

*NMPD* Non-medical use of prescription drugs, *SD* Standard deviation

### SES indicators and substance use, unadjusted analyses

Table 2 presents the relationship of SES indicators to substance use. Compared to those with average or below average perceived family income, participants with perceived above average family income were more likely to report any use of alcohol and monthly RSOD. However, participants with average or below average perceived family income were more likely to report both more daily tobacco use and more than once a week cannabis use than did those with above average perceived family income. The prevalence of other substances use did not differ significantly.

**Table 2:** Substance use outcomes as a function of perceived family income, own education and parents’ education

	Perceived family income					Highest achieved own education level					Parents’ highest achieved education level						
	Average or below average (*N =* 3164, 55.5 %)		Above average (*N =* 2538, 44.5 %)			Obligatory or elementary vocational education (*N =* 2858, 50.1 %)		Higher than obligatory or elementary vocational education (*N =* 2844, 49.9 %)			Obligatory (*N =* 362, 6.3 %)		Secondary (*N =* 2894, 50.8 %)		Tertiary (*N =* 2446, 42.9 %)		
	N/mean	%/SD	N/mean	%/SD	p	N/mean	%/SD	N/mean	%/SD	p	N/mean	%/SD	N/mean	%/SD	N/mean	%/SD	p
Alcohol																	
Any use (past 12 months)	2873	90.8 %	2392	94.2 %	<.001	2653	92.8 %	2612	91.8 %	.162	285	78.7 %	2681	92.6 %	2299	94.0 %	<.001
RSOD monthly or more^a^	1401	44.3 %	1234	48.6 %	.001	1322	46.3 %	1313	46.2 %	.946	104	28.7 %	1338	46.2 %	1193	48.8 %	<.001
Weekly risky drinking^b^	203	6.4 %	152	6.0 %	.507	185	6.5 %	170	6.0 %	.439	16	4.4 %	189	6.5 %	150	6.1 %	.284
Number of drinks/week (mean SD)	7.30	14.74	7.46	10.82	.648	7.46	13.58	7.29	12.69	.619	5.54	25.39	7.43	12.51	7.58	11.05	.021
Tobacco use																	
Any use (past 12 months)	1519	48.0 %	1174	46.3 %	.188	1388	48.6 %	1305	45.9 %	.043	163	45.0 %	1345	46.5 %	1185	48.4 %	.244
Daily use	737	23.3 %	459	18.1 %	<.001	633	22.1 %	563	19.8 %	.029	85	23.5 %	641	22.1 %	470	19.2 %	.015
Cannabis use																	
Any use (past 12 months)	945	29.9 %	799	31.5 %	.189	912	31.9 %	832	29.3 %	.030	87	24.0 %	781	27.0 %	876	35.8 %	<.001
More than once a week	329	10.4 %	214	8.4 %	.012	291	10.2 %	252	8.9 %	.089	33	9.1 %	254	8.8 %	256	10.5 %	.107
Other illicit drugs^c^ use																	
Any use (past 12 months)	193	6.1 %	165	6.5 %	.535	200	7.0 %	158	5.6 %	.025	18	5.0 %	159	5.5 %	181	7.4 %	.010
NMPD^d^ use																	
Any use (past 12 months)	327	10.3 %	280	11.0 %	.396	318	11.1 %	289	10.2 %	.238	45	12.4 %	289	10.0 %	273	11.2 %	.200

*Note.* ^a^Risky single occasion drinking defined as drinking 6 or more drinks on one occasion. ^b^Weekly risky drinking, defined as drinking 21 drinks per week or more. ^c^Ecstasy or cocaine or heroin or magic mushroom. ^d^Sleeping pills or tranquilizer or painkiller or stimulant or antidepressant or beta-blocker

*NMPD* Non-medical prescription drugs, *SD* Standard deviation, *P* p value for chi square test of independence, except for number of drinks/week for which analysis of variance (testing equality of means) was used

Compared to participants who completed more than obligatory or elementary vocational education, those who completed only obligatory school or elementary vocational education reported a higher prevalence of any tobacco use, daily tobacco use, cannabis use, and other illicit drug use. Alcohol and NMDP use were not significantly different.

Participants whose parents had achieved tertiary education were more likely to report any use of alcohol compared to those whose parents had achieved only obligatory and were also more likely to report monthly RSOD and larger weekly alcohol consumption. Daily tobacco use differed across parental education levels, being highest among those whose parents had completed obligatory education only. Cannabis and illicit drug use also differed according to parental education, being highest among participants whose parents had completed tertiary education.

### SES indicators and substance use, adjusted analyses

Table 3 shows the results of the alcohol use outcomes analyses, adjusted for age, living environment and linguistic region. When associations of SES indicators were tested separately, participants with average or below average perceived family income were 35 % less likely to report any use of alcohol and 13 % less likely to engage in monthly RSOD compared to those with an above average perceived family income. Participants whose parents had completed only obligatory education were 73 % less likely to report alcohol use, 54 % less likely to report monthly RSOD and reported drinking significantly fewer standard drinks (1.83, on average) in a week than did those whose parents had completed tertiary education. Participants whose parents completed secondary education were 22 % less likely to report alcohol use and 11 % less likely to report monthly RSOD than did those whose parents had completed tertiary education.

**Table 3:** Regression models of socioeconomic status predicting alcohol use outcomes

	Single indicator model		Fully adjusted model	
Alcohol	OR	95 % CI	OR	95 % CI
Any use (past 12 months)				
Average or below average perceived family income	0.65	0.53, 0.80	0.75	0.60, 0.94
Obligatory or elementary vocational education	0.96	0.78, 1.18	1.01	0.82, 1.25
Parents’ education (ref. tertiary)				
Obligatory	0.27	0.20, 0.37	0.30	0.22, 0.41
Secondary	0.78	0.63, 0.97	0.84	0.67, 1.05
RSOD monthly or more^a^				
Average or below average perceived family income	0.87	0.78, 0.97	0.93	0.83, 1.04
Obligatory or elementary vocational education	0.90	0.80, 1.00	0.92	0.82, 1.02
Parents’ education (ref. tertiary)				
Obligatory	0.46	0.36, 0.59	0.48	0.38, 0.62
Secondary	0.89	0.80, 0.99	0.91	0.82, 1.02
Weekly risky drinking^b^				
Average or below average perceived family income	1.09	0.87, 1.36	1.10	0.87, 1.38
Obligatory or elementary vocational education	1.06	0.84, 1.33	1.06	0.84, 1.33
Parents’ education (ref. tertiary)				
Obligatory	0.75	0.44, 1.27	0.72	0.42, 1.23
Secondary	1.06	0.85, 1.32	1.03	0.82, 1.30
	b	p	b	p
Number of drinks/week^c^				
Average or below average perceived family income	−0.07	.838	0.07	.850
Obligatory or elementary vocational education	−0.09	.808	−0.05	.896
Parents’ education (ref. tertiary)				
Obligatory	−1.83	.014	−1.85	.015
Secondary	−0.14	.694	−0.16	.671

*Note.* All regression models are adjusted for age, living environment and linguistic region. Single indicator model: Alcohol use outcomes on SES indicator tested in separate models. Fully adjusted model: Alcohol use on perceived family income, highest achieved own education, parents’ highest achieved education. ^a^Risky single occasion drinking defined as drinking 6 or more drinks on one occasion. ^b^Weekly risky drinking, defined as drinking 21 drinks per week or more. ^c^Linear regression models, unstandardized regression coefficient are reported

*OR* Odds ratio, *CI* Confidence interval, *P* P value

In the fully adjusted model (when all three SES predictors were entered simultaneously), the observed associations when each SES predictor was tested separately remained significant and of similar magnitude, except for the association between family income and monthly RSOD, and between parents’ secondary education and alcohol use and monthly RSOD. Average or below average perceived family income was independently associated with a lower prevalence of alcohol use. Similarly, participants whose parents had completed obligatory education only were less likely to use alcohol and to report monthly RSOD and consumed fewer drinks per week than did those whose parents had completed tertiary education. Table 4 shows the results of the regressions predicting the other substance use outcomes, using the same models listed in Table 3. When associations of SES indicators were tested separately, average or below average perceived family income was positively associated with daily use of tobacco. Obligatory or elementary vocational education was positively associated with any tobacco use, daily tobacco use, any cannabis use, cannabis use more than once a week, and other illicit drug use. Participants whose parents had completed only obligatory education were 46 % less likely to use cannabis and 40 % less likely to use other illicit drugs, compared to those whose parents completed tertiary education. Similarly, those whose parents completed secondary education were 33 % less likely to use cannabis and were less prone to using other illicit drugs, compared to those whose parents completed tertiary education. Nevertheless they were more prone to report daily tobacco use. There were no significant associations of SES indicators with NMPD use.

**Table 4:** Regression models of socioeconomic status predicting tobacco, cannabis, other illicit drugs’ and NMPD’ use outcomes

	Single indicator model		Fully adjusted model	
Tobacco use	OR	95 % CI	OR	95 % CI
Any use (past 12 months)				
Average or below average family income	1.07	0.96,1.19	1.10	0.99,1.23
Obligatory or elementary vocational education	1.17	1.05,1.31	1.18	1.05,1.31
Parents’ education(ref. tertiary)				
Obligatory	0.84	0.67,1.05	0.80	0.64,1.00
Secondary	0.92	0.83,1.03	0.90	0.80,1.00
Daily use				
Average or below average perceived family income	1.35	1.18,1.55	1.31	1.14,1.50
Obligatory or elementary vocational education	1.33	1.16,1.53	1.31	1.14,1.51
Parents’ education(ref. tertiary)				
Obligatory	1.16	0.89,1.52	1.03	0.78,1.35
Secondary	1.20	1.05,1.37	1.11	0.97,1.28
Cannabis use				
Any use (past 12 months)				
Average or below average perceived family income	0.90	0.80,1.01	1.01	0.90,1.14
Obligatory or elementary vocational education	1.20	1.06,1.35	1.23	1.09,1.40
Parents’ education(ref. tertiary)				
Obligatory	0.54	0.42,0.70	0.53	0.41,0.68
Secondary	0.67	0.60,0.75	0.66	0.59,0.75
More than once a week				
Average or below average perceived family income	1.19	0.99,1.43	1.27	1.05,1.54
Obligatory or elementary vocational education	1.36	1.13,1.65	1.37	1.13,1.66
Parents’ education(ref. tertiary)				
Obligatory	0.77	0.52,1.13	0.68	0.46,1.00
Secondary	0.83	0.69,1.00	0.77	0.64,0.94
12-month use of other illicit drugs^a^				
Average or below average perceived family income	0.89	0.71,1.10	0.96	0.77,1.21
Obligatory or elementary vocational education	1.45	1.15,1.83	1.48	1.18,1.87
Parents’ education(ref. tertiary)				
Obligatory	0.60	0.36,0.98	0.58	0.35,0.97
Secondary	0.74	0.59,0.92	0.74	0.59,0.93
12-month use of NMPD^b^				
Average or below average perceived family income	0.94	0.79,1.11	0.95	0.79,1.13
Obligatory or elementary vocational education	1.15	0.96,1.38	1.16	0.96,1.39
Parents’ education (ref. tertiary)				
Obligatory	1.08	0.77,1.52	1.09	0.77,1.54
Secondary	0.89	0.75,1.06	0.90	0.75,1.08

*Note.* All regression models are adjusted for age, living environment and linguistic region. Single indicator model: Substance use on SES indicator tested in separate models. Fully adjusted model: Substance use on perceived family income, highest achieved own education, parents’ highest achieved education. ^a^Ecstasy or cocaine or heroin or magic mushroom. ^b^Sleeping pills or tranquilizer or painkiller or stimulant or antidepressant or beta-blocker

*OR* Odds ratio, *CI* Confidence interval, *P* P value, *NMPD* Non-medical prescription drugs

In the fully adjusted Model, the observed associations when each SES predictor was tested separately remained significant and were of similar magnitude, except for the association between parents’ secondary education and daily use of tobacco. In addition, the association of average or below average perceived family income and parents’ secondary education with more than once a week cannabis use reached significance.

Those who completed obligatory or elementary vocational education were more likely to report any use of tobacco. The odds of daily tobacco use was higher among participants with average or below average perceived family income and those who had completed only obligatory school or elementary vocational education.

Participants who had completed only obligatory or elementary vocational education were more likely to report any use of cannabis, whereas those whose parents had completed obligatory or secondary education were less likely to use cannabis. Odds of using cannabis more than once a week was higher for those with average or below average perceived family income and for participants who had achieved only obligatory or elementary vocational education but was lower for those whose parents had completed secondary education.

The odds of using other illicit drugs was significantly higher for participants who had completed only obligatory or elementary vocational education but was lower for those whose parents had completed obligatory or secondary education. There were no significant associations of SES indicators with NMPD use.

## Discussion

The first finding of this study was that higher perceived family income and parental education were associated with greater odds for alcohol use, while own education did not seem to be associated with the risk for alcohol use. Higher parental education was associated with a heavier use (monthly RSOD and weekly drinking). Lower perceived family income and own education were associated with greater odds of daily tobacco use. For cannabis, high levels of parental education and lower own education were associated with greater odds of both any use and frequent use (i.e., more than once a week), while lower perceived family income was associated with more frequent use. For other illicit drug use, higher parental education and lower own education were associated with more use, while perceived family income was not related. There was no association of non-medical use of prescription drugs with any of the three SES indicators used in this study. Most of the observed associations and the magnitude of the observed associations remained similar in the fully adjusted model, suggesting independent associations.

Our results confirm what has been shown in other studies. Humensky et al. [13], in a longitudinal study of 20,745 students (ages 12–18) who were subsequently monitored (ages 18–27), showed that individuals with a college educated parent were more likely to engage in monthly RSOD, and cannabis and cocaine use in early adulthood than did those with a high school educated parent. Similarly, individuals with higher household incomes were found to be more prone to monthly RSOD, and cocaine use. In a sample of young adults, Patrick et al. [15] also showed that alcohol and cannabis use in young adulthood were associated with higher parental education.

In a cross-sectional survey conducted in Switzerland, Abel et al. [14] found in a sample of 31,424 males (ages 18–25) that those who had finished obligatory education only were more likely to use alcohol in early adulthood compared to those with more education (OR 1.56). For parental education, the association did not follow a social gradient. Similar to our study, males whose parents had completed only obligatory education were less likely to report risky drinking (OR = 0.5). Additionally, males from households with lower family income were less likely to report risky drinking (OR = 0.81) than were those from wealthier households.

Other studies have also demonstrated unique patterns in the relationship of alcohol to SES indicators [14, 30–33] that are different than those found for other substances. This might be explained by greater social acceptability of drinking, as well as a need for feelings of both belonging and of independence among young adults, that are enhanced by alcohol use [34].

The present study has some limitations. First, the sample consists of males only, and gives no information regarding the association of SES indicators and substance use among women. In addition, this research is based solely on assessments that were self-reported, precluding the use of any biological measures of substance use. Self-reports are potentially influenced by social desirability and recall biases. Also, potential differences between study participants and the source population may limit the generalizability of the study results. In C-SURF, as shown by Studer et al. [17, 18], potential differences in substance use may exist among individuals who agreed to participate in the study and those who were approached but refused to participate in the study. Nonetheless, in that large study the differences observed between consenters and non-consenters were small, even though non-consenters reported higher substance use than did consenters.

When looking at participants own education, a social gradient appears present for most substances. Nevertheless this gradient is no longer present when looking at the participant parents’ education level. Independently of one’s own education level, parental education is likely to play a role in which substances are used and how. This may be explained by a differing perception of alcohol, tobacco and other drugs among parents from different educational backgrounds. Depending on their education level, parents may be more permissive towards a given substance and less towards another. The perception of one’s family economic power was associated with specific substance use patterns (more alcohol use for those perceiving their family has having stronger economic power, more cannabis and tobacco use for those perceiving their family as having weaker economic power). Depending on the SES strata, some substances may be considered more acceptable than others, and the perceived risks related to these substances may be influenced by the SES. Hypotheses for these differences, such as differing perceptions, norms, feelings of belonging, access and availability, etc. should be tested specifically. Studying the mechanisms behind the associations observed in the present study would help develop targeted public health interventions. Nevertheless, the observed differences inform on how to refine preventive intervention for specific population groups.

With regard to generalizability, findings should be interpreted with the Swiss context in mind. The legal age for purchase of alcohol and tobacco, and the illegal status of cannabis and other drugs may differ in other countries. Even though we used the perceived family income as an indicator of SES, the average income in Switzerland is high. The Swiss education system might also differ from what can be found in other countries. Nevertheless, we think our results provide important knowledge: associations between SES and substance use may differ by substance. As such, preventive intervention or public health initiatives have to target alcohol, tobacco and other drugs specifically in the different socio-economic strata.

Notwithstanding the above limitations, the current study has some notable strengths. It was comprised of a large sample of 20-year-old males coming from diverse geographical and socioeconomic backgrounds in Switzerland. It included the assessment of several SES indicators and their associations with different licit and illicit substances, and provided indications on associations of non-medical use of prescription drugs with SES that are seldom studied.

## Conclusions

Economic power (family income) appears to be associated with higher odds of using alcohol, but with lower odds of using cannabis and tobacco daily. Higher parental education level was fairly consistently associated with increased odds of using any substance except tobacco, and is possibly a reflection of varying perceptions regarding alcohol, tobacco, cannabis, and other drug use across different educational backgrounds. For the most part, the education level of participants was negatively associated with the odds of using any substance except alcohol (i.e., the lower the education level, the greater the substance use risk). Thus, the social gradient appears to differ according to the SES indicator and to the substance involved. It would be worthwhile to conduct further investigations that concentrate specifically on the perceptions of various licit and illicit substances among young individuals (as well as among their parents). The findings herein could be used to design better prevention interventions in the future. Appropriate target groups could be assembled, using the knowledge gained thus far regarding SES variables and their relationship to substance use. Notably, alcohol use does not follow the same pattern of association with lower socio-economic indicators as other substances. It should not be grouped with tobacco, cannabis and other drugs. More research is needed on norms, perceptions, acceptability of these substances across SES strata to increase the efficacy of preventive interventions and to match the intervention content to the specifics of each SES stratum. Also, a higher parental education level appear associated with the use of illicit drugs (including cannabis) and binge drinking, which questions how unhealthy substance use patterns are perceived among the more highly educated.

Our results should encourage more research on how various licit and illicit drugs are favorably (or unfavorably) perceived within the general population. Researchers and public health agencies should therefore not take for granted the current popular notion that low SES is necessarily a detrimental factor influencing the consumption of various psychotropic substances.

### Ethics approval

The study was approved by the Ethics Committee for Clinical Research of the Lausanne University Medical School. C-SURF was approved by the Ethics Committee for Clinical Research of the Lausanne University Medical School (protocol number 15/07).

## Abbreviations

SES: socioeconomic status
NMPD: non-medical use of prescription drugs
OR: odds ratio
CI: confidence interval
RSOD: risky single occasion drinking
C-SURF: cohort study on substance use risk factors
